# Language acquisition for deaf children: Reducing the harms of zero tolerance to the use of alternative approaches

**DOI:** 10.1186/1477-7517-9-16

**Published:** 2012-04-02

**Authors:** Tom Humphries, Poorna Kushalnagar, Gaurav Mathur, Donna Jo Napoli, Carol Padden, Christian Rathmann, Scott R Smith

**Affiliations:** 1 Education Studies/University of California, La Jolla, San Diego, CA 92093, USA; 2 Chester F. Carlson Center for Imaging Science, Rochester Institute of Technology, Rochester, NY 14627-899, USA; 3 Department of Linguistics, Gallaudet University, 800 Florida Avenue NE, Washington, DC 20002, USA; 4 Department of Linguistics, Swarthmore College, 500 College Ave, Swarthmore, PA 19081, USA; 5 Department of Communication/9500 Gilman Dr., University of California, La Jolla, San Diego, CA 92093, USA; 6 IDGS, Universität Hamburg, Binderstr. 34, 20146 Hamburg, Germany; 7 National Center for Deaf Health Research, University of Rochester, PO Box 278990, Rochester, NY 14627-8890, USA

**Keywords:** Cochlear implants, Sign language, Deaf children, First language acquisition, Linguistic deprivation

## Abstract

Children acquire language without instruction as long as they are regularly and meaningfully engaged with an accessible human language. Today, 80% of children born deaf in the developed world are implanted with cochlear devices that allow some of them access to sound in their early years, which helps them to develop speech. However, because of brain plasticity changes during early childhood, children who have not acquired a first language in the early years might never be completely fluent in any language. If they miss this critical period for exposure to a natural language, their subsequent development of the cognitive activities that rely on a solid first language might be underdeveloped, such as literacy, memory organization, and number manipulation. An alternative to speech-exclusive approaches to language acquisition exists in the use of sign languages such as American Sign Language (ASL), where acquiring a sign language is subject to the same time constraints of spoken language development. Unfortunately, so far, these alternatives are caught up in an "either - or" dilemma, leading to a highly polarized conflict about which system families should choose for their children, with little tolerance for alternatives by either side of the debate and widespread misinformation about the evidence and implications for or against either approach. The success rate with cochlear implants is highly variable. This issue is still debated, and as far as we know, there are no reliable predictors for success with implants. Yet families are often advised not to expose their child to sign language. Here absolute positions based on ideology create pressures for parents that might jeopardize the real developmental needs of deaf children. What we do know is that cochlear implants do not offer accessible language to many deaf children. By the time it is clear that the deaf child is not acquiring spoken language with cochlear devices, it might already be past the critical period, and the child runs the risk of becoming linguistically deprived. Linguistic deprivation constitutes multiple personal harms as well as harms to society (in terms of costs to our medical systems and in loss of potential productive societal participation).

## Introduction

Medical harm can be due to errors or complications of treatment, but it can also be due to failure to properly inform patients of the information they need to protect their overall health now and in the future. Inappropriate care of the latter type lies usually in unawareness on the part of medical personnel and on lack of coordination among the various medical professionals. Here we discuss medical harm related to the use of cochlear implants with deaf children. Because of lack of training and lack of coordination among professionals, there is a great deal of misinformation about the use of speech and sign language with deaf children who undergo cochlear implantation. Specifically, many medical professionals do not fully understand the ramifications of promoting speech-exclusive approaches and denying sign language exposure to a deaf child before and after implantation.

We describe several harms from the surgery itself, and argue that, ethically speaking, a standard for success should be cochlear implants measured against hearing aids which are less invasive and do not cause permanent damage to the cochlea. In particular, we need studies that show success provided by cochlear implants justifies excluding hearing aids as treatment. We also need more studies that identify predictors of successful implant use as well as which children will benefit from a cochlear implant.

## Background

Whether or not to give a child a cochlear implant has been a point of controversy since cochlear implants were first introduced. The debate is often presented as revolving around the question of whether or not cochlear implants would remove a child from Deaf communities and eventually threaten Deaf communities with extinction [1]. (In writing *deaf*, it is common convention to use a capital "D" when talking about communities that use a sign language as their major language, and "d" when talking about auditory status.)

We don't enter into this debate here. Nor do we enter into a discussion of the ethical questions surrounding cochlear implants, which are complex [2]. Instead, we look at the harms of the implant procedure, risks of hopes for outcomes not realized and leading to depression, economic consequences to society, harmful conflicts of ideology, and other questions associated with performing cochlear implantation surgery. We offer suggestions for remedies where possible.

The number of deaf children who are candidates for cochlear implants is substantial. Sensory neural hearing loss is the most common birth defect globally, occurring in 2 to 3 out of 1000 newborns in developed countries [3-5] and much higher in underdeveloped countries, such as in Nigeria, where we find 28 per 1000 [6]. Post-natal causes of sensory neural hearing loss [7] increase that number, so that by school age, 6 to 7 out of 1000 children have permanent hearing loss [8]. The number of deaf children that are affected is quite large and begs for careful and informed calculation of risk and addressing of harms.

Cochlear implantation has become the standard of care, so much so that in developed countries around 80% of deaf children are implanted, and in some places the figure is even higher [9]. As a result, the harm we address in this paper has already been experienced by a significant number of children.

Most of these children experience harm not only because they do not experience success with the cochlear implant but because they are also not provided with exposure to sign language. Over forty years of research on linguistic and psycholinguistic aspects of sign languages demonstrate that they are human languages acquired and used in the same ways as spoken languages with all the requisite grammatical properties. The lack of awareness of medical professionals that sign language gives deaf children unambiguous and total access to a human language is a source of great harm to many deaf children. With this background, in the following sections we expand on the different areas of concern that we have raised.

## Harm

There are several types of harm associated with cochlear implantation. We focus first on those that follow from the increasingly common practice of health professionals advising, and sometimes insisting, that the family keep the implanted child away from sign language, an act that leads to the harm of linguistic deprivation. This harm is not the result of cochlear implantation itself, but of actions that lead to linguistic deprivation.

### Harms associated with the speech-only approach: Linguistic deprivation

The brain of a newborn is designed for early acquisition of language. Indeed, language acquisition proceeds without explicit training on the part of the already competent language users. Children naturally come to be fluent in whatever accessible language(s) they are surrounded by and exposed to on a regular and frequent basis. The language or languages the child acquires during these early years are called first languages. Around five years of age, the plasticity of the brain begins to gradually decrease. A child who has not acquired a language by that time (often called "the critical period") runs the risk of not acquiring native-like fluency in any language [10-12]. As a result, the child becomes linguistically deprived. Linguistic deprivation occurs rarely among hearing children, and only in the most unusual circumstances, such as in children who have grown up without being surrounded by human language [13], or in children who have been denied language as an act of abuse [14].

The circumstances for deaf children are different. Spoken language is not accessible for many deaf infants and children. This is true even for children who have cochlear implants, because the success rate with cochlear implants is highly variable [15-17]. While many studies of the language and psycho-social development of implanted children conclude that cochlear implants are valuable, other studies of implanted children's language skills in daily communication beyond speech skills within a laboratory setting reveal that an alarmingly large percentage of implanted children are not receiving sufficient benefits and continue to demonstrate weakness in language competence. A closer examination of these studies finds that a significant number of these children do not communicate with ease in a speech-only environment even after years of rehabilitative training [[18-31], among others]. Assuredly, there are spectacular successes that have been reported in the literature cited here, but they do not represent the majority of deaf children, and unfortunately, even those very successful individuals often demonstrate some cognitive difficulties [32-34].

Sign language, on the other hand, is accessible to all deaf children, even to the deaf-blind child since there are tactile versions of sign language [35]. Yet many deaf children are raised in a strictly speaking environment and are not offered sign language until after the age of five or not ever [36-38].

#### Harm to the individual from linguistic deprivation

Not having a solid foundation in any language - not being able to converse with native fluency and with complete ease - this is not all that linguistic deprivation encompasses. Linguistic deprivation carries with it a spectrum of problems beyond strictly language pathologies. Cognitive activities that rely on a firm first language foundation such as mathematics (since symbol manipulation is involved) and the organization of memory are then disordered or disrupted [38,39]. Linguistic deprivation also diminishes one's educational and career possibilities, since the cognitive factor that correlates best to literacy is a foundation in a first language [40-52] - and without literacy one's professional opportunities are highly circumscribed. Additionally, linguistic deprivation leads to psychosocial problems due to the isolation and frustration one experiences from diminished linguistic and cognitive capability. This also results in the inability to express oneself fully, and to easily understand others completely [[53,54], among many]. Clearly, linguistic deprivation constitutes a multi-faceted harm to the individual.

#### Harm to society from linguistic deprivation

Because many deaf people have been linguistically deprived at some level, epidemiological studies of deaf people have revealed some alarming and undesired statistics. Deaf people have a higher rate of illiteracy [55], imprisonment [56,57] and unemployment [58,59]. Illiteracy strongly correlates with high unemployment, poverty and poor health (often due to lack of access to information about good health choices and risky behavior). While poverty has negative effects even for hearing children [60], those effects are multiplied when the child is linguistically deprived, making the child even less likely to be able to participate constructively in society. We also find that deaf children and adults who cannot communicate with those around them are abused more frequently [61-63]. Victims of maltreatment, particularly in childhood, have a higher incidence of mental disturbance and risky behaviors, exacting additional costs on society [64-66]. Given these facts, it is predictable that linguistically deprived deaf people would have a higher incidence of imprisonment - either because they engage in criminal activity, sometimes under coercion [67], or because they cannot participate in defending themselves against accusations of such activities. All of these factors burden society. Further, the loss to society of the potential productivity of all these people is significant. Clearly, linguistic deprivation is harmful not only to the affected individuals, but to the society at large. Next, we discuss how this harm is embedded in current medical acts and practices.

#### Medical acts that harm

There are several medical acts that cause harmful linguistic deprivation for the deaf child.

##### First, failure to inform

Many medical professionals faced with the parents of a deaf newborn or newly deafened child tell them that there are two routes with respect to language and educational choices: the oral route (i.e., access to spoken language only) and the manual route (i.e., using sign language with the child). They then explain that the choice is up to the parents. Presented in this way, parents often think their choice is between their child speaking English or communicating using signs that are not understood by most people in the society. Unfortunately, to date, most professionals do not realize there is another choice, a bimodal choice (i.e. using sign language while at the same time promoting English/speech development).

Some professionals explicitly frame the parents' choice as a cultural choice [68]. The child either grows up as a deaf person immersed among people who hear, or grows up like those deaf people they see signing on the streets or in the deaf programs they may have visited. The problem with this choice is that this choice is often based on a stereotypical view of deaf people and not on an adequate portrayal of well-functioning, well-adjusted deaf people who might also use sign language. Unfortunately, at this time, only a few medical professionals have the knowledge or training to give better advice. The result of this uninformed or misinformed advice is often unintentional harm to the child and family.

The vast majority of deaf infants (approximately 96%) are born to hearing parents, who often know very little about sign language or Deaf communities [69]. These parents are in a state of vulnerability, grieving the loss of a normally hearing child and fearing what the future may hold (or not hold) if their child cannot speak like a hearing child [70]. They might view sign as an inferior choice or a last resort [71,72] and not fully understand that sign language is a human language with the linguistic complexity and expressiveness of spoken language. They might also fear their child will be stigmatized if they use a sign language [73]. Furthermore, they might be afraid of trying to learn a new language at their age [74]. In the absence of relevant information, many parents opt for the speech-only route because, without appropriate advice and information, they do not understand the risks of linguistic deprivation.

Medical professionals who work with deaf children and their families need to step forward and assume the responsibilities that the situation places on them. If they intend to give advice to their deaf patients about language development, they need to inform themselves about how some principles of first language acquisition might be more important for deaf children. Visual language skills can contribute to deaf children's language development (one can teach phonology to deaf children through sign language without using any sounds, for example). A deeper understanding of this would lead medical professionals to tell parents of deaf children that sign language offers them accessible language and prescribe this as a medical necessity.

##### Second, misinforming and coercing behavior counter to the child's welfare

Many medical professionals, faced with vulnerable parents of deaf newborns or newly deafened small children, offer the speech-only route as entirely different from the bimodal route. Frequently, they will urge parents to keep the child away from sign, offering the paradoxical justification that signing will be so "easy" that the child will lose motivation to learn to speak, and that the child can always learn a sign language later precisely because it is so easy [75]. This advice to avoid sign language is comprehensive and extends through both home and school [76-78]. Sometimes, families are encouraged or actually required to sign an agreement to this effect [79]. As a result, some parents will demand in their child's Individualized Education Plan at school that their child be removed from any access to sign language and be educated with speech/hearing only [80]. Cochlear implant protocols that prohibit the use of alternative accessible language are causing linguistic deprivation in deaf children who do not acquire a first language by early childhood.

These professionals are acting under misapprehensions. There is no evidence to back up the claim that sign languages are easier to learn than spoken languages or that if a child learns a sign language, that child will lose motivation to work at gaining speech skills. Many young hearing children are exposed regularly and frequently to multiple accessible languages and acquire them both (or all) with little effort. Bilingualism and multilingualism have a range of cognitive as well as professional and personal advantages for anyone, deaf or hearing [81]. If the deaf child is truly finding the spoken language accessible, there is no reason to expect that child to abandon acquiring the oral language simply because they are also acquiring a sign language. On the other hand, if the oral language is not accessible, then it is crucial that the deaf child acquire a sign language, since without it, the child will experience linguistic deprivation.

Certainly, the issue of accessibility of oral language with respect to the deaf child is a nuanced one. All implanted deaf children, for example, need intensive therapy or training in order to have a chance to access spoken language [82]. And the question still remains whether the amount of access to speech that the implant might bring will translate into substantial qualitative and quantitative access to language, substantial enough to acquire it. This is because a cochlear implant bypasses the ear canal and transforms auditory information into electrical impulses that are directly delivered to the cochlea [30,83]. As a result, accessing spoken language for the deaf child, takes effort and intervention. Still, children put out effort to learn new activities all the time - from those that are mostly motoric in nature such as walking or riding a bike, to more cognitively based activities such as drawing a tree or reading and singing. If a child experiences progress at and benefit from an activity, that gratification is sufficient to motivate further work at the activity. It is generally only in the face of no progress that children will quit at activities crucial to daily living.

The deaf child who signs experiences gratification, and the deaf child who makes progress in producing speech and in both speech-reading and aural comprehension also experiences gratification. There is no reason to think that these deaf children, particularly those who are making progress, cannot experience even greater gratification with bimodal language exposure and gain competency at both.

##### Third, abnegation of trust

Many deaf children raised in speech-only environments receive little to no accessible language. In the face of lack of progress or disappointingly low progress, parents may try to remain optimistic. They might be resourceful and try new rehabilitative techniques. They might be patient and encouraging to the child, hoping that it's just a matter of time. Medical professionals often condone and foster these parental behaviors because they, too, are invested in the success of the implant. Too often, the family does not realize that the child is experiencing severe difficulties in language development until the child is falling behind hearing peers in settings outside the home, often at school [84].

Some of these parents lose faith in their medical professionals and sometimes extend that lack of faith to medical professionals in general. They may not know where to turn. They suffer from indecision and stress that they might be failing their child. The result is a delay in facing the child's problem [84], often until the critical opportunity for language acquisition has passed. The abnegation of trust causes this delay, and such a delay is highly detrimental [43,71,85].

### Harms associated with cochlear implant surgery

All surgeries have risks. Cochlear implant surgeries are no exception. Complications associated with cochlear implant surgery include injury to the facial nerve, necrosis and breakdown of the flap, injury to hair follicles, and improper electrode placement [86]. Post-surgery complications include infection under the flap and in the middle ear as well as meningitis [86]. 40% to 74% of patients experience post-operative vertigo that can last for years [87,88]. Further, many times the apparatus fails due to traumatic damage or technical failure and requires repeated surgery or surgeries with all the same associated risks [89].

Additionally, the cochlear implant surgery typically disables the cochlea [30], although new hybrid cochlear implant apparati stimulate only the basal end of the cochlea where the high frequency hearing has deteriorated, preserving the residual low-frequency hearing. Since the implanted ear might well have had some residual hearing, if the cochlear implant does not offer language access to the child, then the surgery has, in fact, had a contrary result to its very intention. Hearing aids do not present the same risk.

The harms of cochlear implant surgery are actually increasing. More and more children are being binaurally implanted, which means a second surgery with all its risks and loss of residual hearing in both ears [90]. Additionally, the results with binaural implants are just as variable as with a single implant with no clear benefits having been established for the binaural choice although it is often claimed that two implants are better than one [91]. Further, while in the early days of implantation children were not considered candidates unless it was clear that they were receiving very little language input auditorily, today children are implanted even when they recognize up to 30% of sentence material [92]. Since 30% is a better recognition rate than many children have post implantation, these children actually might be losing ground with respect to speech skills.

## Remedies

We have seen medical harm from cochlear implants due to the faiure to inform properly, the failure to protect the overall health of the child, and complications in treatment - in other words, cochlear implants can cause most of the major types of harm that medical procedures can cause [93]. There are clear remedies for harm caused by linguistic deprivation. We outline those first. Then we turn to a discussion of how to limit harm caused by implant surgery itself.

### Remedies to prevent linguistic deprivation

There are several remedies that the medical profession can enact by making sign language available.

#### First, recommend sign language

All children need and deserve an accessible language. This is a biological need. The medical profession must protect the health of deaf children by setting a foundational goal of prevention of linguistic deprivation, which can be achieved via sign language. Deaf children need to be given an opportunity to interact with other Deaf peers (i.e. signing children) during their childhood. This ensures that they develop social and communicative abilities. Additionally, for expanded professional and social opportunities, the medical profession can and should also recommend training in spoken language skills. However, such recommendations should never exclude sign language because sign language prevents linguistic deprivation. This is a reliable and implementable remedy to reduce the risk and the harm of linguistic deprivation.

The cochlear implant team must protect the implanted child by demonstrating ways that the family can raise the child with sign language. They should direct the family to sign language classes if the family has not already done this, and to support services that will help introduce the family to the Deaf community. They should require continued sign language exposure, both regular and frequent, through the elementary school years to help ensure that deaf children will have good language skills regardless of their success with the cochlear implant. Sign language skills are essential in successful use of interpreters in educational, professional and social settings, especially those requiring communication in large and complex interactions (such as a public presentation). They should understand and help the family understand that using a sign language is not an inferior method of communication, but that sign languages are complex, expressive languages in which any matter can be communicated, no matter how technical or nuanced [94]. In order to give knowledgeable advice in this regard, schools and continuing education programs for health professionals should include courses on language acquisition for deaf children as well as the status of sign language as a natural language and Deaf communities as rich in culture and history which a family can look forward to exploring [95,96]. The traditional deference to parental autonomy needs to be mitigated when parents' knowledge about language acquisition in deaf children is not sufficient to make well-informed health decisions for their deaf children [97].

#### Second, adjust expectations from cochlear implants

Unbridled optimism pervades the scene of hearing loss in the developed world today, as evidenced by the fact that 80% of deaf children in many developed countries are implanted [9]. We are inclined to view any new technology as an advance in leaps and bounds, thus we think cochlear implants will soon effectively bring "hearing" to implanted people. Unfortunately, this optimism is not only unfounded, it is also unrealistic. While we have witnessed computers go from gigantic mechanisms with limited power to miniscule mechanisms with awe-inspiring power, the technology of cochlear implants is not comparable to the technology of computers. Cochlear implants do not involve only progress in technology; they also involve a biological interface between technology and the human brain. Not only must we find the right way to encode and deliver interpretable information to the brain, we must then find the right way to train the brain to decode and interpret that information. This is not easy. Hearing aids have been around much longer than cochlear implants; indeed, hearing aid technology has improved vastly, yet there are still thorny challenges [98]. Cochlear implants have been implanted in adults with the US Food and Drug Administration approval since 1972 and in children starting in 1985 with clinical trials [99], so if the matter were simply technology, we'd have expected dramatic changes in implant success rates by now. Studies are not reporting substantial increases in success rates, especially in language development. The real challenge is the interface of brain and technology.

As a result, we are required to objectively evaluate benefits against risks. Medical professionals must begin to expect this particular road - the cochlear implant road - to be a long, hard journey, and they should not give families the false impression that technology today has advanced to the point where spoken language is easily and rapidly accessed by implanted children. Once we are sensible about our expectations, parents will then better understand why they need to give their deaf children a sign language and they will then be more likely to comply with medical recommendations to that effect.

#### Third, coordinate delivery of medical services to the deaf child across the relevant health professionals

At this time, a primary care physician who might receive an abnormal newborn hearing screening is likely to send the family of a deaf child to an audiologist and have no further contact with that family regarding this issue. The audiologist then might send the child to a surgeon for a cochlear implant, who likewise has no further contact with the family. The surgeon sends the child to a rehabilitation team that may or may not work with the child for years. Medical insurance in the United States may not cover post-implant rehabilitation sessions, resulting in withdrawals and poor CI outcomes. Unfortunately, information is too rarely shared among these professionals. Often, there is not a single medical professional who is looking at the chain of treatment interventions and who can respond in a timely and appropriate manner to language development issues. Here, coordination of information and efforts will reduce the likelihood of delay in recognizing the warning signs of linguistic deprivation and of failing to respond before the critical period for language acquisition has passed. This is a critical area of need because it goes to the heart of accountability and responsibility of medical professionals. Given the highly variable and limited success of the cochlear implant protocol from evaluation to surgery to training and education, there must be a way for professionals involved to be aware of and be accountable for the consequences of their actions. To date, most research studies do not acknowledge concerns such as the ones we have outlined.

#### Fourth, study successful CI users and learn from them over a period of time

At this point we know that success among CI users is highly variable. It is difficult to compare studies of success rates because the standards of identifying success vary widely. In addition, these subjects comprise socially and culturally heterogeneous groups [100]. Regardless, we must find a way to compare studies so that we can know what factors do correlate with cochlear implant success, especially language development and communication outside of laboratory settings, and better understand who is a good candidate for them. The is a first step in getting a better grip on the benefits versus risks situation that we face today and in making realistic implant decisions at all levels based on reliable data.

### Remedies to limit harm from cochlear implant surgery

The medical profession is continually trying to find improvements in cochlear implant technology and surgery [101]. Nevertheless, the problem is the surgery itself. The only real way to limit harm from cochlear implant surgery in an ethical way is to make sure that only the children who have an excellent chance of gaining more benefit from a cochlear implant than from a hearing aid be implanted.

## Conclusion

Inititally, the burden was on the cochlear implant industry to show the benefits of surgery. As recently as 2001, a Council of Europe report that evaluates studies of deaf children's language acquisition from various countries quotes a Finnish document that says, "No study has yet shown that a congenitally deaf child learns spoken language by means of the implant so that he/she can cope with normal communication outside the laboratory" [[102], page 33]. As a result of considering the material from all the input countries, this council recommended all deaf children be taught sign language as they learn to read and write in the ambient spoken language, and it called for more studies on the efficacy of cochlear implants. The findings of that report are still largely true: cochlear implant "stars" are visible, but they are few and far between. Though medical studies rarely address this, economic motivations behind the cochlear implant industry compounded by unrealistic optimism regarding understanding of the interface between technology and the human brain might be promoting earlier and broader use of cochlear implants in deaf children without adequate long-term studies to support these actions. The result is that the cochlear implant industry has taken the upper hand and the burden to prove harm has now shifted to those who urge caution and support sign language as a plan for timely first language acquisition. Because there is so much we cannot predict about what implants do, and so much we already know about what they don't do, we believe that no child should be implanted unless there is a very strong chance that child will have excellent oral communication skills as a result of implantation and rehabilitation. And because we know that sign language acquisition from an early age leads to normal language acquisition, every deaf child should be raised with sign language as protection against the harm of late first language acquisition. Counseling families to make a strict choice between modalities is inadvisable [103]. Bimodalism - reading and writing in the ambient spoken language combined with a sign language - is advisable. Developing a child's speech skills is also advisable [103], but while cochlear implants are presently the prevalent technology, hearing aids may deliver the same benefits without the risks.

## Authors' contributions

All authors contributed equally to the drafting of the manuscript. All read and approved the final manuscript.
